# Intra-Specific Diversity of *Leishmania major* Isolates: A Key Determinant of Tunisian Zoonotic Cutaneous Leishmaniasis Clinical Polymorphism

**DOI:** 10.3390/microorganisms10030505

**Published:** 2022-02-25

**Authors:** Hanene Attia, Manel Rabia Sghaier, Aymen Bali, Fatma Zahra Guerfali, Sadok Chlif, Chiraz Atri, Nabil Belhaj-Hamida, Amor Zaatour, Adel Gharbi, Afif Ben-Salah, Koussay Dellagi, Dhafer Laouini

**Affiliations:** 1 Institut Pasteur de Tunis, LR16IPT02, Research Laboratory on Transmission, Control and Immunobiology of Infections (LTCII), 13 Place Pasteur, BP 74, Tunis-Belvédère 1002, Tunisia; hanene.attia@pasteur.tn (H.A.); manel.sghaier@pasteur.tn (M.R.S.); aymen.bali@pasteur.tn (A.B.); fatma.guerfali@pasteur.tn (F.Z.G.); sadok.chlif@pasteur.tn (S.C.); chiraz.atri@pasteur.tn (C.A.); Nabil.BelHadjHmida@pasteur.tn (N.B.-H.); amor.zaatour@pasteur.tn (A.Z.); adel.gharbi@pasteur.tn (A.G.); afif.bensalah@pasteur.tn (A.B.-S.); Koussay.Dellagi@pasteur.fr (K.D.); 2 Campus Universitaire Farhat Hached, Université Tunis El Manar, Romana-Tunis 1068, Tunisia; 3 Department of Medical Epidemiology, Institut Pasteur de Tunis, 13 Place Pasteur, BP 74, Tunis-Belvédère 1002, Tunisia; 4 Department of Family and Community Medicine, College of Medicine and Medical Sciences, Arabian Gulf University, Road 2904 Building 293, Manama 329, Bahrain

**Keywords:** *L. major*, zoonotic cutaneous leishmaniasis (ZCL), virulence, clinical polymorphism

## Abstract

The clinical expression of zoonotic cutaneous leishmaniasis (ZCL) caused by *Leishmania* (*L.*) *major* parasites has a broad spectrum ranging from asymptomatic infection to self-limited cutaneous sores or severe disease. In concert with the host immune responses, the vector variability and the number of bites, genetic variation between *L. major* isolates might impact on the clinical output of the disease. We investigated herein the intra-specific variability of *L. major* field isolates independently of host or vector factors and then tried to correlate parasite variability to ZCL severity in corresponding patients. Several assays were applied, i.e., in vivo pathogenicity of promastigotes in a BALB/c mice model, resistance/sensibility to complement lysis, in vitro growth kinetics, and expression of different lectins on the promastigote surface. Combining all these parameters allowed us to conclude that the resistance to complement lysis and PNA/Jacalin lectins binding to parasite surfaces are important markers of parasite virulence. These factors correlate significantly with clinic polymorphism of ZCL and modestly with genetic micro-heterogeneity, a characteristic we previously revealed with a MLMT profile.

## 1. Introduction

About 20 *Leishmania* (*L.*) species are considered as pathogens for humans and cause a group of diseases known as leishmaniasis [1]. Among them, cutaneous leishmaniasis (CL) is a major public health problem worldwide. Intriguingly, different clinical manifestations of CL caused by the same *Leishmania* species have been described. For instance, uncommon lesions caused by *L. major* reported in Sudan are difficult to recognize as *Leishmania* infection and are difficult to treat [2]. In Iran, CL cases caused by *L. major* were reported as polymorphic. Indeed, the clinical presentation of cases varied from typical lesions to atypical ones including erythematous volcanic ulcers, multi-infections, lupoid, diffuse, eczematous, verrucous, dry, and nodular lesions [3]. In Tunisia, *L. major* is the most prevalent dermotropic *Leishmania* species and causes zoonotic cutaneous leishmaniasis (ZCL) [4]. It is a polymorphic disease, even within a small endemic focus, with various clinical presentations ranging from asymptomatic infection to benign self-limited cutaneous sore(s) or to more extensive lesion(s) causing severely disfiguring scars [5].

The role played by each partner involved in the parasitic cycle (the vector, the host, and the parasite) and how they interact together determine the clinical expression of the disease. The specific aim of this work is to study the impact of parasite variability on the clinical polymorphism of the disease. Hence, parasite intra-specific variability might contribute to parasite pathogenicity and virulence. Accordingly, several parasite molecules have been described as virulence factors. For example, surface lipophosphoglycan (LPG) phosphoglycans prevent complement-mediated lysis, serve as a ligand for receptor-mediated endocytosis, and inhibits the microbicidal oxidative burst and the phagosome–endosome fusion. On the other hand, zinc metalloprotease gp63, the major cell surface glycoprotein of *Leishmania* promastigotes, has been described to be actively implicated in the degradation of host macromolecules, serves as a ligand for the macrophage, and protects the parasite against complement-mediated lysis [6,7]. Kebaier et al. reported a correlation between in vitro growth of promastigotes and their virulence [5].

We previously reported, using multi-locus microsatellite typing (MLMT) with 10 highly informative and discriminative markers, the genetic structure of Tunisian *L. major* isolates collected from patients living in different old and emerging foci of Central Tunisia [8]. Phylogenetic reconstructions showed that one locus (71AT) had a 58/64-bp bi-allelic profile with an allele linked to emerging foci. Interestingly, isolates collected from the newly emerging foci were either homozygotes or heterozygotes for the 58 bp allele, whereas those obtained from the old foci were homozygotes for the 64 bp allele. What is worthy of note is that most isolates with the 58 bp allele induced a severe disease in humans [8].

In order to study the intra-specific variability of *L. major*, as one of the key factors of ZCL clinical polymorphism and virulence, we report here the experimental pathogenicity in a BALB/c mouse model, the in vitro resistance to complement lysis, the in vitro growth kinetics, and the expression of carbohydrates on the parasite surface of 18 Tunisian *L. major* isolates obtained from the field, and correlated the results to their clinical manifestation in human patients from whom they were isolated. Results suggest that differences in resistance to complement lysis and differences in the expression of galactose-associated glycoconjugates on the parasite surface, referred to here as parasite virulence biomarkers, may be a determinant for the virulence of *L. major* parasites in human hosts.

## 2. Materials and Methods

### 2.1. Ethics Statement

The study protocol, consent and assent forms and procedures were reviewed and approved by the Institut Pasteur de Tunis Ethical Review Board. Patients, from whom parasites were obtained (or their parents or legal guardians in case of minors), provided written informed consent for the collection of isolates and their subsequent analysis. Animal experiments were performed in compliance with the directive 86/609/EEC of the European parliament and the council on the protection of animals used for scientific purposes, in agreement with the guidelines of International Guiding Principles for Biomedical Research Involving Animals and with the approval of the Ethical Review Board of the Institut Pasteur de Tunis (Ref#07/07).

### 2.2. Reagents and Animals

A total of four lectins used for this study were purchased from Sigma: Peanut agglutinin (PNA), Artocarpus integrifolia agglutinin (Jacalin), soybean agglutinin (SBA), and Lens culinaris agglutinin (LcH). To perform complement lysis experiments, we used human AB serum collected from healthy donors and commercialized by Promo Cell. The serum was divided into two parts, treated or not in 56 °C during 30 min. To preserve the complement-proteins activity, the serum was conserved at −80 °C until use. BALB/c female mice were obtained from the specific pathogen-free animal-breeding facility Janvier (France) and maintained in a conventional animal facility at Institut Pasteur de Tunis, Tunisia.

### 2.3. Study Area, Sample Collection from Human Patients, and Follow-Up

Eighteen strains of *L. major* were isolated from fresh lesions of patients living in five villages (i.e., Mnara, Mbarkia, Dhouibet, Ksour, Msadia) in the governorates of Sidi Bouzid and Kairouan, Central Tunisia, an endemic area of cutaneous leishmaniasis caused by *L. major* [9]. Promastigotes forms were obtained and harvested within the shortest time after promastigotes growth, as described previously [10], deep frozen, and conserved at −80 °C. Patients were followed up during 180 days on a weekly basis to monitor the evolution of lesions, through time, until cure. Lesion severity was then calculated and corresponds to the integral of the approximation of the evolution of the lesion’s area over time, reported from onset to cure dates (Supplementary Table S1). ITS1-PCR and RFLP were performed, as previously described [8,11,12,13], to confirm that all isolates belong to *L. major* species. 

### 2.4. Clinical Severity Score

The date of onset $\left(t_{0}\right)$ was obtained from the patient by asking about the time he/she first noticed the ZCL lesion. At that time, we assumed that the lesion was too small and considered to have a null size $\;(L_{0}=0,\;W_{0}=0)$. This measurement was taken at the $\;Day_{1}$ visit during which the length $\left(L_{1}\right)$ and width $\left(W_{1}\right)$ of the lesion were measured. The lesion boundary was defined, most of the time, following the lesion induration. When the latter was absent, the lesion was reduced to ulcerated area. A continuous line represented the ellipse that approximated the ZCL lesion’s area. The major and minor diameters of this ellipse represented the length (*L*) and width (*W*) of the lesion, respectively. During the following visits $(t_{i},\;i>0$) at 15-day intervals from $Day_{15}$ to $Day_{90}$ and at $\;Day_{180}$, subsequent measurements $\left(L_{i},\;W_{i},\;i>0\;\right)$ were taken using the same procedure.

The area of the lesion at $t_{i}$, namely $A_{i}$, was computed using the following equation:

(1) $$A_{i\;}=\frac{\pi }{4}L_{i}W_{i}\;\;,\;i\geq 0$$

where:

(1) $L_{i}=\mathrm{length}\;\mathrm{of}\;\mathrm{the}\;\mathrm{lesion}\;\mathrm{at}\;\mathrm{time}\;t_{i}.$
(2) $W_{i}=\mathrm{width}\;\mathrm{of}\;\mathrm{the}\;\mathrm{lesion}\;\mathrm{at}\;\mathrm{time}\;t_{i}.$

The set $(t_{i},\;Ai)\;i\geq 0$ represents an approximation of the evolution of the lesion’s area over time, denoted A(t). The integral of the latter, from onset to cure dates, represents the disease burden caused by the lesion and is called severity in this paper.

### 2.5. Metacyclic Form Purification

Experiments were conducted using metacyclic purified parasites. This step allowed working with homogenous parasites to allow comparability between experiments when indicated. Briefly, when the stationary phase was reached, the metacyclic promastigotes were purified by a negative selection with peanut agglutinin (PNA). The PNA^−^ and PNA^+^ fractions were separated by density gradient centrifugation, and the formers were washed and diluted at the desired density.

### 2.6. Animal Infection and Monitoring of the Disease Pattern

For each of the 18 *Leishmania* isolates included in the study, a group of six eight-week-old BALB/c mice was infected with 2 × 10^6^ metacyclic promastigotes. The parasites were suspended in 50 µL of 1 × PBS and injected in the left footpad of the animal. The right footpad was considered as an uninfected control.

From the second week of infection, lesion development was assessed weekly by measuring the diameters of the right and left pads by the same operator and using a vernier caliper. Lesion development was then assessed by measuring the thickness diameter difference between the infected and non-infected footpad of every mouse (mm).

At the end of the experimental protocol, mice were sacrificed and the infected and contralateral footpad, the draining lymph node, and the spleen of each mouse were excised and precisely weighed. Parasite load from infected mice was determined by limiting-dilution method following a protocol described elsewhere [14,15]. After 7–10 days of culture at 26 °C, viable parasites were checked microscopically and their load was then determined as the reciprocal of the highest dilution at which promastigotes could be grown in culture at 26 °C and detected by microscope [15].

### 2.7. In Vitro Complement Lysis

Promastigotes were collected on the sixth day of culture, washed and pelleted by centrifugation, then resuspended in RPMI-20% FCS at 10^8^ cells per mL and distributed in 96-well plates at 100 μL/well. In vitro complement lysis and the percentage of parasite survival were monitored for each isolate as we previously described [8]. 

### 2.8. In Vitro Growth Kinetics of L. major Promastigotes

Promastigotes of the stationary stage were collected on the sixth day of culture. The parasites were washed three times in RPMI medium and then suspended in RPMI/10% FCS at a concentration of 1 × 10^6^ parasites per mL. For each isolate, eight 25 cm² flasks containing 7 mL of the culture were prepared and incubated at 26 °C. One flask of each culture was counted daily at the same time, during eight days, by three different operators. The mean and the standard deviation of the results generated by the three operators, for every day, were used to draw the regression curve of the 18 isolates included in the study.

### 2.9. Lectin-Binding Assays

ELISA plates were treated with poly-L-lysine at a final concentration of 10 µM per well during one hour at room temperature. For every parasite isolate, and for every lectin, 10^6^ promastigotes per well were plated over three columns of the poly-L-lysine-treated ELISA plate.

After saturation with PBS 1% BSA, seven serially diluted biotinylated-lectin concentrations (from 50 to 0.753 µg/mL) were used. Every isolate was tested in triplicate for each of the four lectins. Two conditions were tested as controls. In the first condition, promastigotes were incubated without lectins. In the second one, promastigotes were incubated with the maximal concentration of the biotinylated lectin in the presence of its specific inhibitor sugar. The different lectins tested, their specificity, and the concentration of the inhibitor sugar are summarized in Supplementary Table S2.

The parasites were then incubated with the extrAvidin-peroxidase. After adding the TMB peroxidase substrate (SIGMA), optic density (OD) was measured at 450 nm and the binding index was calculated as following:

Binding index = OD with parasites − OD without parasites.

### 2.10. Statistical Analysis

All statistical tests were performed using PRISM software. To measure correlation between the parameters tested, we used the Spearman correlation that does not require a large sample size and makes no assumption about the distribution of the values, as the calculations are based on ranks instead of the actual values. We chose a two-tailed *p* value and a confidence interval of 95%. To measure the significance of differences between the compared groups, we used the Mann–Whitney test, a nonparametric test that compares the distributions of two unmatched groups.

## 3. Results

### 3.1. Experimental Pathogenicity in BALB/c Mice

For each one of the 18 tested *Leishmania* isolates, a group of six BALB/c mice was infected. Footpad sizes were weekly measured during eight weeks using the caliper. The sizes of the contralateral footpads of each mouse were also measured on the same day. For the strain 0796, we had to stop the measurements and mice were sacrificed at the sixth week for ethical reasons, as the infected footpad was very inflamed and could be lost.

**Figure 1:**
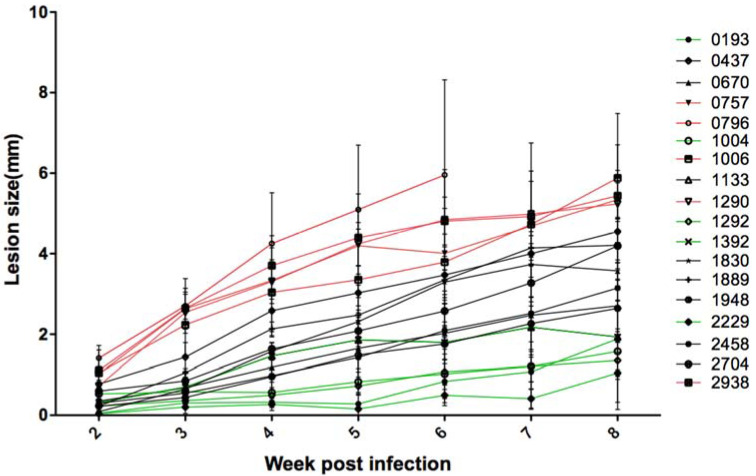
Lesion size of the BALB/c mice footpads infected with the 18 *L. major* isolates. Lesion size was monitored using the caliper every week during 8 weeks. The lesion measurement was stopped on the sixth week for a single isolate (0796) for ethical reasons. For each isolate, a group of six mice was infected. The mean and standard deviation of the six mice were presented for each isolate. The results are representative of one experiment out of two performed with six mice for each isolate.

The results (Figure 1) showed a heterogeneous evolution of the lesions during the infection course, depending on the parasite isolate. Lesions reached the eighth week of infection with different sizes from 1 to 6 mm. Five of the 18 isolates (2229, 1292, 1004, 0193, and 1392, represented in green, Supplementary Figure S1A) caused small lesions less than or equal to 2 mm in the eighth week of infection. Five isolates (0796, 0757, 1006, 1290, and 2938, represented in red, Supplementary Figure S1C) caused large lesions greater than 5 mm. The remaining isolates caused medium lesions (between 2.7 and 4.5 mm of width, represented in black, Supplementary Figure S1B).

**Figure 2:**
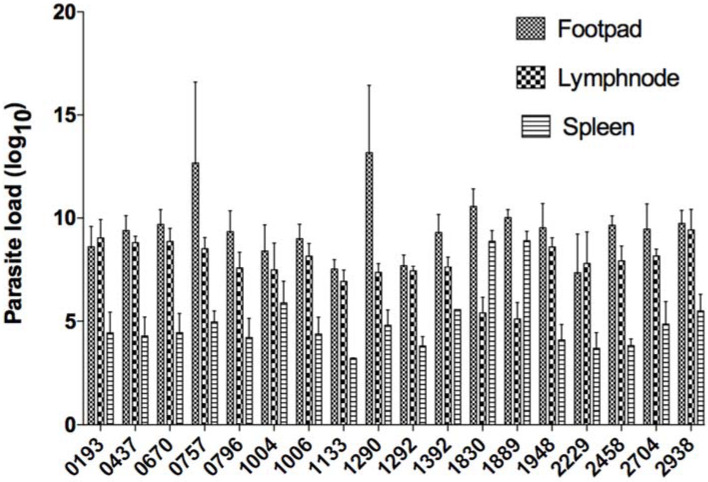
Parasite load in whole infected footpads, spleens, and lymph nodes of BALB/c mice infected with the 18 *L. major* isolates. For each one of the 18 isolates included in the study, a group of six BALB/c mice were sacrificed on the eighth week after infection (excluding isolate 0796 that was sacrificed on the sixth week after infection) and parasite load in three organs (infected footpad, lymph node draining the infection site, and spleen) was determined by limiting dilution assay. The results are expressed as the means ± standard deviations of the log_10_ dilutions of infected footpads, draining popliteal lymph nodes, or spleens that were positive for *L. major* promastigotes. The results represent triplicates from six individuals in each group. The results correspond to those obtained from one representative experiment out of two performed.

**Figure 3:**
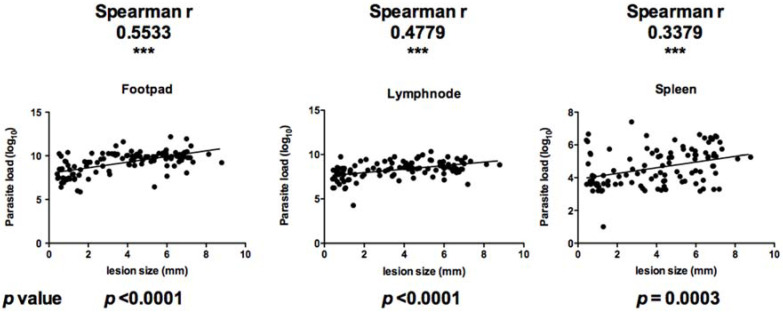
Correlation between the size of the lesion developed in the infected footpad, the lymph node draining the infection site, and the spleen on the one hand, and the parasite load in the corresponding organs. Correlation was measured using the non-parametric correlation coefficient Spearman r. The significance of the correlation is evaluated with the *p* value. *** indicate highly (*p* < 0.001) statistically significant values.

At the end of the protocol, mice were sacrificed and infected feet, draining lymph nodes, and spleens were excised and weighed. Parasite load was then monitored using limiting-dilution (Figure 2). Parasite load in infected feet correlated positively with lesion size (Spearman r value of 0.5533 and *p* value less than 0.0001). In the draining lymph node, parasite load correlated positively with lesion size with a Spearman r value of 0.4779 and *p* value less than 0.0001. Parasite load in the spleen correlated positively with lesion size (Spearman r value of 0.3379 and *p* value of 0.0003) (Figure 3).

### 3.2. In Vitro Resistance to Complement Lysis

Resistance/sensitivity to complement lysis of the 18 *L. major* isolates was tested by incubating stationary phase promastigotes in the presence of fresh or heat inactivated human serum. After contact with complement proteins, the number of living parasites was monitored using MTT reagent (Sigma). The percentage of viability corresponds to the ratio of living parasites in the presence of fresh serum to the number of living parasites in the presence of heat inactivated serum.

In the presence of 32% of fresh serum and 20 mM EDTA, 100% of the promastigotes belonging to every tested isolate resisted complement lysis. Such controls indicate that lysis that occurred in different experimental conditions is specifically due to complement proteins.

**Figure 4:**
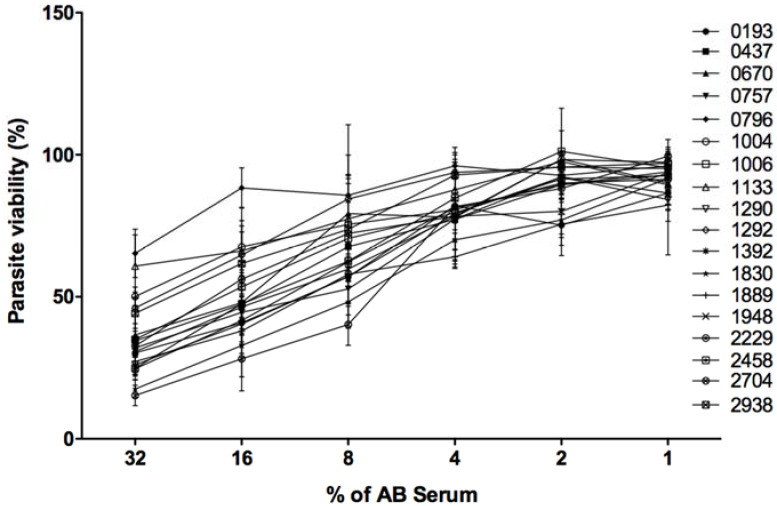
Resistance/sensitivity of 18 *L. major* isolates to complement lysis. Parasites were incubated with different concentrations of human serum (*x*-axis). Viability was measured using the MTT assay. Parasite viability is the percentage of viable parasites in the presence of complement proteins versus viable parasites in the presence of heat deactivated serum. The mean and the standard deviation of three experiments are presented for each isolate.

As shown in Figure 4, the 18 parasite isolates reacted differently to complement proteins. When the human serum was used at 32%, three isolates (0796, 1133, and 1004) were the most resistant to complement lysis with respective viabilities of 65, 60, and 50%. Six isolates (2704, 1830, 1392, 2458, 1290, and 1889) were the most sensitive to complement lysis with respective viabilities of 15, 17, 24, 24, 26, and 27%. The remaining isolates were moderately resistant to complement lysis with a viability percentage ranging between 30 and 50%.

When human fresh serum was used at 16 and 8%, 0796 remained the most resistant isolate to complement lysis showing 88 and 85% of viability, respectively. The isolate 2704 was the most sensitive to complement lyses showing only 28 and 40% of viability, respectively.

All tested isolates showed more than 90% of viability when the fresh human serum was used at a very low concentration (1%).

### 3.3. In Vitro Growth Kinetics

**Figure 5:**
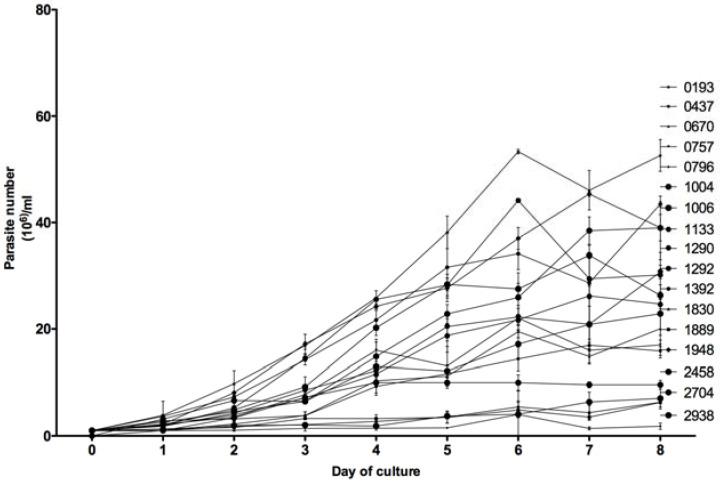
In vitro growth kinetics of the 18 *L. major* isolates. Parasites were cultured at the initial concentration of 1 × 10^6^ parasites per mL and counted every day during 8 days by three different operators. The mean and standard deviation are presented for each isolate. The results correspond to one representative experiment out of at least three ones performed for each isolate.

For each of the 18 tested *Leishmania* isolates, the number of parasites in an in vitro promastigotes culture (1 × 10^6^) was counted every day by three different operators during eight days. As shown in Figure 5, the in vitro growth kinetics of the 18 isolates were heterogeneous but allowed us to distinguish two groups. Eight isolates (0193, 0437, 0670, 0757, 0796, 1889, 2704, and 2938), barely reached 20 × 10^6^ parasites/mL at the eighth day of culture, whereas the 10 other isolates reached higher concentrations; the highest one observed with isolate 2229 (57 × 10^6^ parasites per mL).

**Figure 6:**
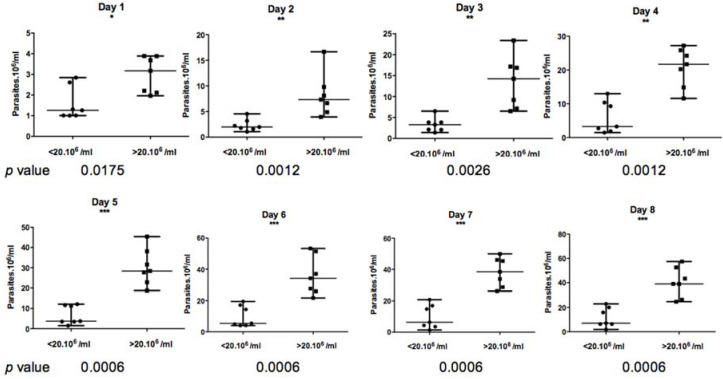
Non-parametric two-tailed *t* test measuring the difference between the two *L. major* isolate groups showing differential in vitro growth kinetics. The first group of isolates reached less than 2 × 10^7^ parasites per mL on the eighth day of culture, whereas the second group exceeded 2 × 10^7^ parasites growth per mL. *, ** and *** indicate significant (*p* < 0.05), strongly (*p* < 0.01) or highly (*p* < 0.001) statistically significant values respectively.

Based on the Mann–Whitney non-parametric test (Figure 6), the difference between these two groups appeared to be statistically significant from day 1 to day 8 of culture, with a *p* value less than 0.01.

### 3.4. Lectin Agglutination Profile of L. major Isolates

We tested the agglutination of the 18 *L. major* isolates with four biotinylated lectins of different specificities (PNA, Jacalin, LcH, and SBA) at seven different concentrations (50, 25, 12.5, 6.25, 3.12, 1.56 and 0.753 µg/mL). In the absence of lectins, no signal was detected for the 18 strains included in the study. When promastigotes were incubated with different biotinylated lectins at 50 µg/mL and their specific inhibitor sugar, the binding decreased drastically, indicating the specificity of the test.

**Figure 7:**
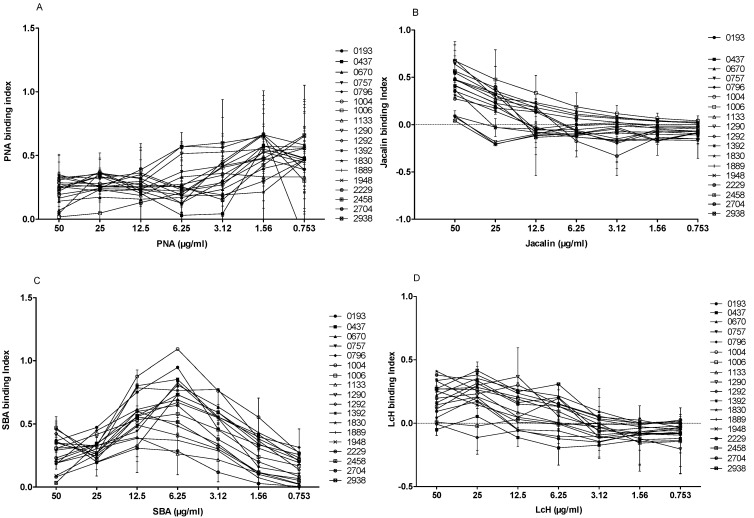
Binding profile of the four lectins PNA (**A**), Jacalin (**B**), SBA (**C**), and LcH (**D**) against 18 *L. major* isolates. The mean and the standard deviation of binding index for at least three experiments are presented for each isolate.

PNA lectin is specific to galactose, a main component of LPG and an important *Leishmania* antigen. As shown in Figure 7A, the difference of agglutination between isolates was evident when PNA was used at 50, 25, or 12.5 µg/mL. At 50 µg/mL of PNA, six isolates (1006, 0193, 1133, 0670, 0757, and 2458) bound weakly (binding index < 0.2). The remaining isolates showed a PNA interaction with a strong binding index.

Similar to PNA, Jacalin is a lectin that can also bind to galactose. When Jacalin was used at 50 µg/mL, four isolates (1290, 1292, 1392, and 2458) bound weakly (Figure 7B), whereas 3 out of 18 *L. major* isolates (0670, 0757, and 1006) bound more strongly than others (binding index > 0.6).

SBA lectin binds specifically to the N-acetyl-D-galactosamine. Interestingly, the SBA binding profile to 18 promastigote isolates (Figure 7C) showed a binding peak at 6.25 µg/mL. Two isolates (1133 and 2704) showed the lowest specific index (<0.3), whereas two other isolates (0193 and 1004) showed the highest specific index (0.947 and 1.092, respectively).

We finally tested the LcH lectin that specifically binds *α*-D-mannose/*α*-D-glucose. Interestingly, three isolates (0437, 1292, and 2458) did not bind to LcH, while the other isolates showed a relatively low (0.1–0.3) binding index (Figure 7D).

### 3.5. Correlation between In Vitro Resistance to Complement Lysis and ZCL Severity

In order to link the in vitro resistance/sensitivity to complement lysis with *Leishmania* parasite virulence, a non-parametric correlation test measuring the correlation between resistance to complement lysis of the different *L. major* isolates included in the study and the disease severity in corresponding human patients was used.

As severity scores were available in only 15 isolates among the 18 included in the study, the Spearman correlation coefficient was calculated using only results from those isolates.

In the presence of 32% of fresh human serum, the correlation between resistance to complement lysis of the 15 isolates and the disease severity in corresponding human patients was significantly positive (Spearman r = 0.7179, *p* value = 0.0026).

This result indicates that the more resistant the *L. major* isolate is to complement lysis, the greater its ability to induce a severe disease in human patients.

### 3.6. Correlation between Experimental Pathogenicity and ZCL Severity

When analyzing experimental pathogenicity observed at each week and severity scores obtained in patients from whom corresponding isolates were obtained, no statistically significant correlation was shown at any stage of experimental pathogenicity with measured human severity. Indeed, and even at weeks 6, 7, or 8, no statistical significance was found. Spearman values were as follows: r = 0.08361 (*p* = 0.7763) for week 6, r = −0.04402 (*p* = 0.8465) for week 7, and r = 0.06052 (*p* = 0.8443) for week 8 of experimental pathogenicity.

### 3.7. Correlation between Experimental Pathogenicity and In Vitro Growth

When analyzing experimental pathogenicity and in vitro growth kinetics results, we noticed that isolates growing slowly in in vitro conditions induce the most severe lesions in the BALB/c mice model. For instance, isolates 0796, 1889, and 2938 that showed the lowest in vitro growth kinetics not exceeding 7 × 10^6^ parasites/mL at day 8 of culture (1.8, 6.2, and 7 parasites per mL, respectively) induced the largest lesions in BALB/c mice, exceeding 4 mm (5.9, 4.2, and 5.4 mm, respectively).

On the other hand, isolates 2229, 1292, and 1392 that showed the most important in vitro growth kinetics exceeding 40 × 10^6^ parasites/mL at the 8th day of culture (57.4, 46, and 43 × 10^6^ parasites/mL respectively) induced the less severe lesions in BALB/c mice (1.16, 1.8, and 1.9 mm respectively).

These observations were confirmed using the non-parametric two-tailed correlation test measuring the linear correlation between these two parameters with a confidence interval of 95% and including the 18 isolates of the study. Hence, our results clearly showed a significant negative correlation between lesion size induced in infected animals and in vitro growth kinetics (Spearman correlation coefficient r= −0.5545 and *p* value = 0.0169), indicating that the more virulent the *L. major* isolate is in the experimental BALB/c mice model, the less it grows in vitro.

### 3.8. Correlation between Lectin Binding Capacity and Disease Severity in Humans

The glycocalyx, expressed by *Leishmania* parasites, plays a key role in host–parasite interaction and in protecting the parasite within the hostile mammalian host environment. To investigate the potential contribution of glycoconjugates surface expression of *Leishmania* parasites to the clinical expression of the disease, we used the Spearman non-parametric test to measure correlation between binding capacity of 15 isolates to lectins and disease severity in patients from where parasites were obtained.

The correlation between PNA binding capacity on the promastigote surface and disease severity scores in patients was found to be significantly negative when the lectin was used at 25 µg/mL (Spearman r = −0.5201, *p* value = 0.0469) or at 12.5 µg/mL (Spearman r = −0.4571, *p* value 0.0867). Interestingly, the correlation between Jacalin binding capacity on the parasite surface and disease severity scores in patients was significantly positive when it was used at 50, 25, or at 12.5 µg/mL (Spearman correlation coefficients r = 0.7714 (*p* = 0.0008), r = 0.6679 (*p* = 0.0065), and r = 0.5179 (*p* = 0.048), respectively).

No correlation was found between disease severity and the binding of SBA and LcH lectins.

Although PNA and Jacalin are both known to bind specifically to galactose, our results showed that severity of the lesion in human patients negatively correlates with PNA binding capacity on promastigotes but is positively correlating with Jacalin binding capacity.

### 3.9. Correlation between MLMT Profiles and Binding Capacity to Lectins or Disease Severity

**Figure 8:**
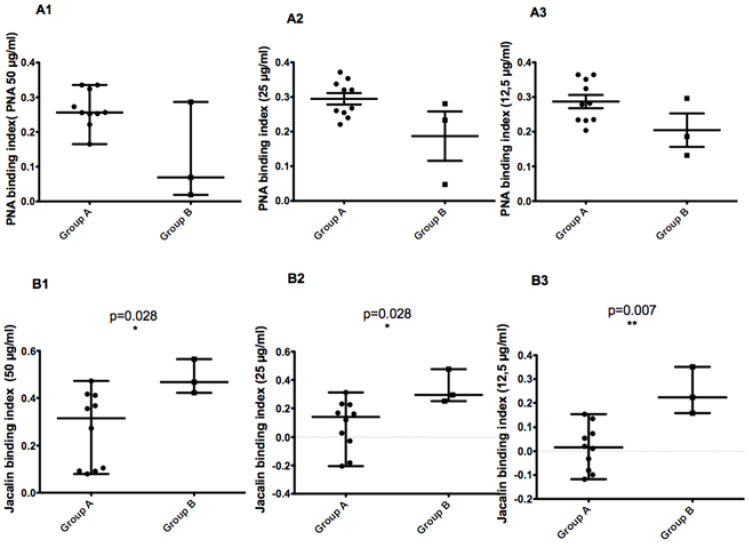
Non-parametric two-tailed Mann–Whitney test measuring the difference in glycoconjugates expression on the promastigote surface between two *L. major* isolates groups showing different MLMT genetic profiles. The first group of isolates (Group A) is composed of parasites with the 68 bp-71AT. The second group of isolates (Group B) are parasites with the 58 bp-71 AT allele. The binding indices of different PNA lectin concentrations (50, 25, and 12.5 µg/mL) for the promastigotes belonging to the two groups are represented in (**A1**–**A3**), respectively. Binding indices of different Jacalin lectin concentrations (50, 25, and 12.5 µg/mL) for the promastigotes belonging to the two groups are represented in (**B1**–**B3**), respectively. * and ** indicate statistically significant (*p* < 0.05), and strongly statistically significant (*p* < 0.01) values respectively.

**Figure 9:**
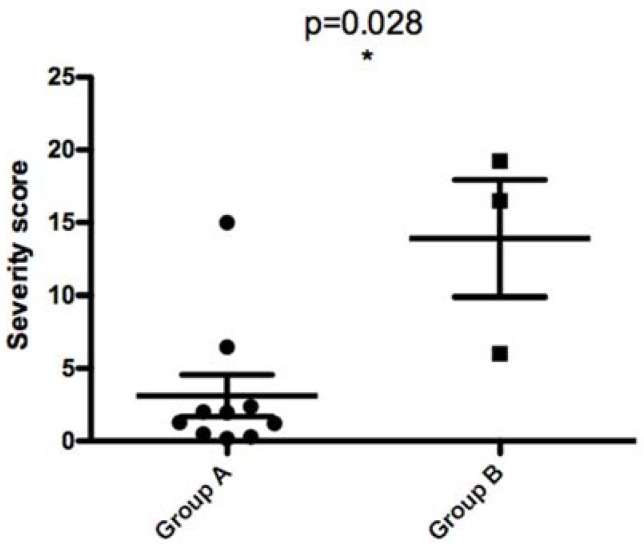
Non-parametric two-tailed Mann–Whitney test measuring the difference in disease severity (severity score as measured in patients) between two *L. major* isolates groups showing different MLMT genetic profiles. The first group of isolates (Group A) is composed of parasites with the 68 bp-71AT allele, whereas the second group of isolates (Group B) is composed of parasites with the 58 bp-71 AT allele. * indicates statistically significant values.

As previously described [8], MLMT typing of 35 *L. major* Tunisian isolates revealed a micro-heterogeneity related to focus history. We also reported that isolates bearing the 58 bp allele were more resistant to complement lysis. In the present work, we used a non-parametric two-tailed test to measure the correlation between the MLMT profile of 13 isolates on the one hand and glycoconjugates expression on the promastigote surface (Figure 8) or disease severity (Figure 9) in the corresponding patients on the other hand.

When comparing parasites with the 68 bp-71AT (Group A) allele to those with the 58 bp-71 AT allele (Group B), promastigotes of the latter bound less to PNA lectin and more to Jacalin lectin. Although the results are reproducible in all tested PNA lectin concentrations, there is no significant statistic difference between the two groups. For Jacalin, the difference between the two groups was statistically significant at all tested lectin concentrations (*p* values of 0.028, 0.028, and 0.007 for 50, 25, and 12.5 µg/mL, respectively).

When comparing parasites with the 58 bp-71 AT allele and those with the 68 bp-71AT allele according to the disease severity they induced in patients, we found a statistically significant difference between the two groups (*p* value of 0.028). This result clearly demonstrates that parasites with 58 bp-71 AT allele seem to induce a more severe disease in human patients (Figure 9).

## 4. Discussion

One characteristic of *L. major* is the diversity of the lesion profiles it induces. This clinical polymorphism is essentially related to the immune status of patients but also to the diversity of intrinsic parasite factors. The main goal of this study was to investigate intra-specific diversity of *L. major* isolates using diverse parasite markers and evaluate their impact on the clinical polymorphism of ZCL due to *L. major* in Tunisia.

We investigated a population of individuals living in an endemic area of ZCL in Tunisia. This population was followed for three ZCL transmission seasons. We were able to collect 18 *L. major* isolates with their corresponding severity scores induced in human patients.

We previously reported, using MLMT typing, a micro-heterogeneity related to focus history. Indeed, parasites with the 58 bp-71 AT allele were detected only in new emerging foci where ZCL is more severe than what was observed in old foci. We also reported that isolates bearing the 58 bp-71 AT allele were more resistant to complement lysis [8].

We studied, herein, the intra-specific functional diversity of the 18 isolates using different parasite virulence markers (in vivo pathogenicity of parasites in BALB/c mice model, binding of different lectins to the promastigote surface, in vitro growth kinetics, and resistance/sensibility to complement lysis) and their potential correlation with clinical polymorphism of ZCL.

The 18 isolates showed different experimental pathogenicity profiles in BALB/c mice, from weakly to moderately or highly pathogenic isolates causing important lesions. The two parameters used to measure parasite pathogenicity (lesion size and parasite load) correlated positively and significantly. Our results corroborate the heterogeneity of previous results from our laboratory describing the experimental pathogenicity of Tunisian and Middle East *L. major* isolates, in a BALB/c mice model [5]. We also tested the in vitro growth kinetics of the isolates included in this study. The 18 isolates were widely heterogeneous showing slow, medium, or fast in vitro growth kinetics.

Unexpectedly, most pathogenic isolates in the BALB/c mice model showed the slowest in vitro growth kinetics and vice versa. Indeed, our results are different from what is classically assumed. For instance, it was reported that the most pathogenic *L. major* isolates in the BALB/c mice model showed the faster in vitro growth kinetics [5], suggesting that pathogenicity in the BALB/c mice model positively correlates with in vitro growth kinetics, while we are suggesting the opposite. It is worth noting that Kebaier et al. used only five isolates to correlate the pathogenicity in a BALB/c mice model and the in vitro growth kinetics. The difference in our different sampling size could explain such contradictory results. It is known that parasite fitness increases with transmission, whereas higher virulent organisms decrease their fitness because it damages their food supply (the host). If both transmission and virulence are coupled, the parasite fitness will then depend on a balance between the costs of increased virulence and the benefits of high transmission [16].

By applying appropriate statistical tests, our results indicate that there is no significant correlation between ZCL severity and experimental pathogenicity of isolates or their in vitro growth kinetics. This result can be argued even partially by the heterogeneity of human immune responses, while the experimental model used consanguine mice. Indeed, human anti-parasitic and immune responses to the same isolate should be different from one individual to another, depending on their genetic and immune background. In addition, several lines of experimental evidence indicate that the infection site and injected parasite number influence the development and the progression of the disease [2].

We also tested the expression of some glycoconjugates on *L. major* promastigote surface using an in-house lectin binding assay. Similar to other protozoa, the membrane surface components of *Leishmania* play an essential role in the host–parasite interaction. Membrane glycoconjugates are important for protecting the parasite from the hostile environment in the phlebotomy vectors as well as the mammalian host. Specific receptors expressed on the *Leishmania* surface, essentially carbohydrates, are directly implicated in parasite survival once introduced into the host and then involved in parasite infectivity [17].

As different lectins recognize specific sugars, these molecules have been widely used to characterize and locate carbohydrates exposed to different protozoa [18] and *Leishmania* species [19], to isolate membrane carbohydrates, to study developmental differences and meta-cyclogenesis of *Leishmania* species including *L. major* [14,20,21,22], *L. donovani* [23,24], and *L. braziliensis* [14], and to compare glucantime-sensitive isolates to resistant ones [17,25].

Few studies have investigated the differential exposure of glycoconjugates on the membrane surface of strains belonging to the same species of *Leishmania*. Some authors studied the functional diversity of five Indian *L. tropica* isolates and reported a correlation between infectivity in a BALB/c mice model, agglutination with lectins, and promastigotes binding to macrophages [26]. In our study, we measured glycoconjugates expression on *L. major* promastigote surfaces using lectins with different sugar specificities: PNA that binds specifically to β-D-galactose-(→3)-dGalNAc, Jacalin lectin that binds specifically to Gal/GalNAc, SBA binding specifically to N-acetyl-D-galactosamine, and LcH binding specifically to *α*-D-mannose/*α*-D-glucose. In the present work, we developed a quantitative agglutination test based on the ELISA principle. The presence and the expression level of membrane carbohydrates were determined using lectin-biotin/streptavidin-peroxidase. Only PNA and Jacalin lectins showed clear differences between the tested isolates. We found, using the Spearman non-parametric statistical test, a positive and significative correlation between ZCL severity and Jacalin binding while PNA binding showed a negative and statistically significative correlation. The loss of PNA binding on *L. major* promastigote surfaces has been previously reported. In fact, using agglutination with six lectins including PNA and SBA, Sacks et al. compared surface carbohydrates of different developmental promastigote stages [21]. They reported the loss of agglutination of approximately 50% of stationary-phase *L. major* promastigotes by PNA and RCA lectins. SBA, which binds to terminal *α*- or β-linked dGalNAc, and Con A, which binds to D-glucose and D-mannose, did not distinguish between log- and stationary-phase promastigotes. The authors also tested WGA and UEA I lectins that did not agglutinate to promastigotes of any stage [21]. These results corroborate ours as we did not find any significant correlation between SBA binding on the different *L. major* isolates’ surfaces and disease severity in corresponding human hosts. Sacks et al. suggested that changes in PNA agglutination were restricted to *L. major* promastigotes as no difference in the agglutination of log- and stationary-phase *L. donovani* promastigotes by PNA could be detected. On the other hand, loss of PNA-binding sites has been described for *L. donovani* promastigotes after conversion into amastigotes [23]. The decrease of agglutination of PNA, which binds preferentially to β-D-galactose-(→3)-dGalNAc, with infectious stage of *L. major* promastigotes might reflect a decrease in the expression of subterminal D-galactose residues on these parasites [21]. According to our results, we also suggest that the significant decrease of PNA binding to *L. major* isolates inducing severe disease in corresponding human patients might reflect a decrease in the expression of subterminal D-galactose residues on these organisms implicating a galactose-containing glycoconjugate on the parasite surface.

In order to further prove the virulent character of parasites bearing the 58 bp-71 AT allele we compared, in the present work, glycoconjugates expression in parasites bearing the 58 bp-71 AT to those bearing the 68 bp-71 AT using PNA and Jacalin lectins.

Parasites with the 58 bp-71 AT allele bound less to PNA lectin. Although the difference between the two groups was not significant, it was reproducible in all tested lectin concentrations. Interestingly, parasites with the 58 bp-71 AT allele bound more to Jacalin lectin. The difference between the two groups was significant with all tested lectin concentrations. The latter results are quite interesting as parasites with the 58 bp-71 AT allele behave like virulent isolates inducing severe ZCL: binding less to PNA and more to Jacalin. In addition, parasites with the 58 bp-71 AT allele induced a more severe disease in human patients than those with the 68 bp-71AT allele. The difference between the two groups was statistically significant. Regarding the latter results, we could hypothesize that genetic intra-specific heterogeneity among *L. major* Tunisian isolates revealed by MLMT might have an indirect impact on parasite virulence and consequently on clinical polymorphism of ZCL. It is worth noting that galactose is a component of a major molecular determinant of *Leishmania* promastigote surface LPG. *Leishmania* parasites are covered by a complex glycocalyx whose glycoconjugate components and LPG *L. major* backbone is galactosylated with 1–4 β-Gal residues and oftenly terminated with Ara [6,25,26].

McConville et al. showed that, during metacyclogenesis, the LPG molecule of *L. major* is modified [7]. At the stationary stage, when promastigotes are the most virulent, there is an increase in repetitive units from 14 to 30 units, a decrease in βGal or Galβ1-3Galβl- glucidic endings abundance, and an increase in the abundance of repetitive units without glucidic endings or with Ara*α*l-2 Galβ1-endings [7]. The latter modifications of LPG induce a dramatic decrease in galactose exposition on the promastigote surface. Even if present, the terminal galactose molecules are hidden with the elaborated ramifications of LPG making them inaccessible to PNA lectin, arguing for the decrease in PNA agglutination of metacyclic promastigotes compared to procyclic ones [27,28]. In the present work, we proved a negative correlation between PNA binding to stationary stage promastigotes and disease severity in corresponding patients. We can, thus, suggest that the promastigote stage of isolates inducing severe disease is more virulent and expose on their surface LPG molecules structurally similar to those exposed by metacyclic infecting promastigotes.

On the other hand, although PNA and Jacalin both belong to the family of galactose-binding lectins, statistical tests allowed us to demonstrate a positive correlation between Jacalin binding to stationary stage promastigotes of the tested isolates and disease severity in corresponding patients. The differential binding properties of PNA and Jacalin lectins have been previously reported [21]. Jacalin is unable to agglutinate the *Leishmania* species of the *donovani* complex. In addition, Jacalin recognizes galactose (ß 1-3) N-acetyl-galactosamine, a structure identical to the receptor for PNA. However, PNA require this O-linked oligosaccharide to be devoid of sialic acid whereas Jacalin will bind to the fully sialylated disaccharide. Jacalin, in combination with PNA, may provide important information on glycoproteins containing O-linked oligosaccharides. Determining the reasons for this difference between the results obtained by the two lectins will help to better understand the metacyclogenesis of *Leishmania* parasites.

We also studied resistance of different *L. major* isolates to complement lysis. Once activated by parasite infection, the complement system plays a key role in recognizing and killing *Leishmania* parasites. Indeed, metacyclic promastigotes need to survive exposure to the complement, an antimicrobial innate immune defense system between inoculation and macrophage phagocytosis phases [29]. Many studies that investigated the interaction of *Leishmania* parasites and the complement system, suggested that the infective form of the parasite is resistant to complement lysis. Metacyclic promastigotes of different *Leishmania* species, such as *L. chagasi* [30] and *L. shawi* [29], would be adapted to the complement system before their contact within the hostile environment in a vertebrate host. They are thought to survive complement-mediated lysis through at least three mechanisms: proteolytic inactivation of the complement cascade, fast release of membrane attack complexes, and a glycocalyx structure that causes membrane attack complex assembly too distal to the parasite membrane. Three highly abundant and developmentally regulated parasite surface macromolecules could be involved in this resistance: major surface protease gp63 that cleaves complement C3b into an iC3b-like form able to block the complement cascade while still functioning as an opsinogen able to facilitate parasite phagocytosis via receptor CR3, LPG, and promastigote surface antigen (PSA, or gp46) that may increase resistance to complement-mediated lysis [30,31].

The infective stage of *Leishmania* parasites is not only resistant to complement lysis, but their interaction could also enhance intra-cellular parasite survival. Resistance to complement lysis is then an important marker of different *Leishmania* species’ virulence. Differential resistance of procyclic and metacyclic stages of the parasite [28], as well as parasites belonging to different *Leishmania* sub-genera [32], have been studied. To our knowledge, there are no reports describing differential resistance of *L. major* isolates to complement lysis and its relationship with clinical polymorphism. The 18 Tunisian tested isolates revealed an important heterogeneity ranging from sensitive to resistant isolates. However, such results have to be examined cautiously since we used stationary parasites instead of metacyclic purified parasites, whose use might reduce the observed heterogeneity. 

Interestingly, we could establish a positive correlation between ZCL severity in humans and resistance of corresponding isolates to complement lysis. Similar to the expression of galactose-linked glycoconjugates on parasite surfaces, the increase of resistance to complement lysis of the different *L. major* isolates could be in coherence with the changes in LPG molecules. In fact, the implication of LPG as a molecular determinant of *Leishmania* promastigotes resistance to complement lysis has been reported [28]. In mammalian hosts, LPG inhibits complement lysis by preventing the membrane attack complex C5b-9 insertion into the promastigote membrane. LPG is also a ligand for complement and mannose receptors allowing parasite endocytosis by macrophages [6]. For these reasons, it could be important to study the relationship between complement lysis and galactose expression. We revealed a negative correlation between PNA binding to the promastigote surface of the different isolates and their resistance to complement lysis. We also found a positive correlation between Jacalin binding to the promastigote surface of the different isolates and their resistance to complement lysis. It is important to note here that almost all of our experiments were confined to the promastigote stage, which is inoculated on human skin by a sand fly bite, encounter the innate immune system, infect intradermal polynuclear cells and macrophages, and differentiate into intracellular amastigotes stage. Hence, the impact of our results, although quantitatively and qualitatively influencing the behavior of the following intracellular stage, is mainly limited to the promastigote stage.

In conclusion, we studied intra-specific diversity of 18 Tunisian *L. major* isolates using different parasite virulence markers; in vivo pathogenicity of promastigotes in BALB/c mice model, resistance to complement lysis, in vitro growth kinetics, and expression of different lectins on the promastigote surface. The different assays showed an important heterogeneity within Tunisian *L. major* isolates. Among tested parameters, and given the statistical correlation tests, we can conclude that resistance to complement lysis and PNA/Jacalin binding to parasite surface are important markers of parasite virulence correlating with clinic polymorphism of ZCL due to *L. major* in Tunisia.

As a whole, our results strongly suggest that a virulent strain of *L. major* that induces a severe disease in human hosts is more resistant to complement lysis in vitro, and the promastigote stage binds more strongly to Jacalin lectin but less to PNA.

## Supplementary Materials

The following supporting information can be downloaded at: https://www.mdpi.com/article/10.3390/microorganisms10030505/s1, Figure S1: Lesion size of BALB/c mice footpads infected with low, middle, and high pathogenic *L. major* isolates; Table S1: List of the *L. major* Tunisian isolates, the villages where they were isolated from, and the calculated severity score in corresponding human patients; Table S2: Different tested lectins, their specificities, and the final concentrations of their specific inhibitor sugars.
