# Supplementary material for: Intra-Specific Diversity of Leishmania major Isolates: A Key Determinant of Tunisian Zoonotic Cutaneous Leishmaniasis Clinical Polymorphism
Source: Microorganisms. 2022 Feb 25;10(3):505. doi: 10.3390/microorganisms10030505 (PMC8955835; doi:10.3390/microorganisms10030505)
Supplement: Supplementary file 1 [file microorganisms-10-00505-s001.zip › microorganisms-1562847-supplementary.pdf]

Supplementary Materials

Table S1. List of the *L. major* Tunisian isolates, the villages where they were isolated from, and the calculated severity score in corresponding human patients. Mnara and Mbarkia villages are considered as old foci, whereas Ksour, Dhouibet, and Msadia are considered as newly emerging foci. Isolates were harvested between October and March, the period in which the lesions of most individuals infected during the ZCL transmission period appear.

Isolate ID	Origin	Severity Score in the Human Patient
0193	NA	NA
0437	Mnara	2.013
0670	Mbarkia	19.956
0757	Mbarkia	6.46
0796	Mbarkia	14.991
1004	Dhouibet	19.233
1006	Dhouibet	6.009
1133	Dhouibet	16.498
1290	Dhouibet	0.28
1292	Dhouibet	2.374
1392	Dhouibet	1.277
1830	Msadia	1.963
1889	Msadia	1.216
1948	Msadia	0.484
2229	Ksour	0.174
2458	Ksour	NA
2704	Ksour	NA
2938	Ksour	3.93

NA: not available.

Table S2. Different tested lectins, their specificities, and the final concentrations of their specific inhibitor sugars.

Lectin	Origin	Specificity	Inhibitor Sugar	Final Concentration of Inhibitor Sugar
PNA	*Arachis hypogaea*	Galactose	D-(+)-Galactose	2 M
Jacalin	*Artocarpus integrifolia*	α-D-galactopyranoside	D-(+)-Galactose	2 M
LcH	*Lens culinaris*	α-D-mannose<br>α-D-glucose	D-(+)-Mannose	2 M
SBA	*Glycine max*	N-acetyl-D-galactosamine	N-acetyl-D-galactosamine	1 mM

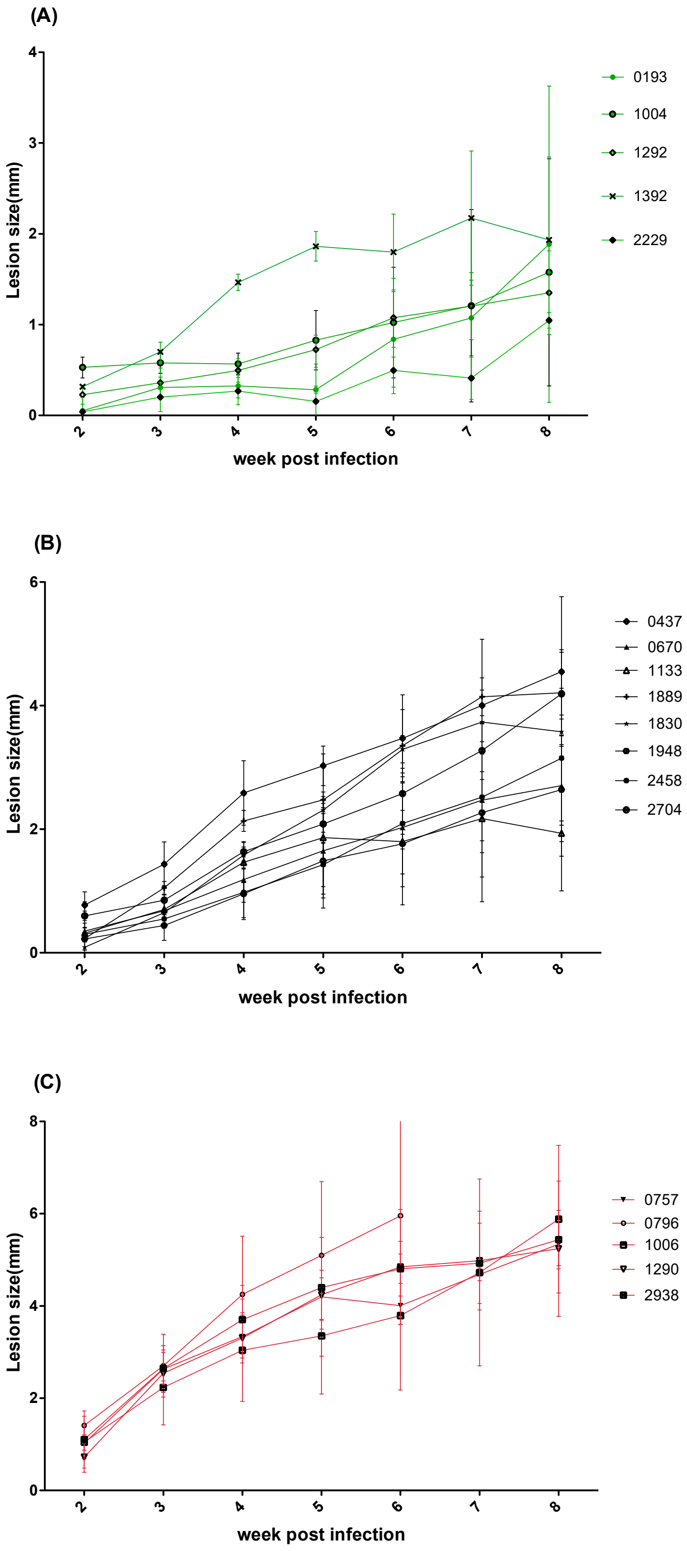

Figure S1. Lesion size of BALB/c mice footpads infected with low (green, panel **A**), middle (black, panel **B**), and high (red, panel **C**) pathogenic *L. major* isolates. Lesion size was monitored using the caliper every week during 8 weeks. For each isolate, a group of six mice was infected. The mean and standard deviation of the six mice were presented for each isolate. The results correspond to those obtained from one representative experiment out of two performed with six mice for each isolate.
